# Plant Tissue Culture and Secondary Metabolite Production Volume II

**DOI:** 10.3390/plants12223862

**Published:** 2023-11-15

**Authors:** Kalina Danova, Ilaria Marchioni, Laura Pistelli

**Affiliations:** 1 Institute of Organic Chemistry with Centre of Phytochemistry, Bulgarian Academy of Sciences, Acad. Georgi Bonchev Str., bl. 9, 1113 Sofia, Bulgaria; 2 Department of Food and Drug, University of Parma, Parco Area delle Scienze 47/A, 43124 Parma, Italy; 3 Department of Agriculture, Food and Environment, University of Pisa, Via del Borghetto 80, 56124 Pisa, Italy

Secondary metabolites play a key role in the communication of the plant organism with the everchanging biotic and abiotic stimuli of its versatile environment. Thus, plant secondary metabolites possess specialized physiological functions in signaling stress and defense reactions. Their chemical type and production capacity are defined by the plant species, its genotype, physiology and developmental stage, as well as the external factors of its environment [1]. These innate functions of secondary metabolites within plant organisms also make them a relevant source of physiologically active principles for mammalian organisms, underlying their reactive oxygen species (ROS) scavenging activity, the ability to chelate toxins and heavy metals and exert toxicity on both external pathogens, as well as on compromised cells of the human organism itself, etc. Thus, obtaining sustainable sources of secondary metabolite production, as well as performing fundamental research to improve our understanding of their biogenesis in the plant organism, are vital for their utilization in human health. The controlled conditions of plant in vitro cultures are a well-manageable experimental system on a different degree of plant tissue differentiation. This method makes it possible to source secondary metabolites for fundamental studies, preserve plant germplasm for natural biodiversity conservation and deliver raw material for industrial application, as well as for food, cosmetic and pharmaceutical purposes. We are pleased to present this Special Issue of *Plants* entitled “Plant Tissue Culture and Secondary Metabolites Production Volume II”. The present collection is a continuation of the previously published Special Issue of “Plant Tissue Culture and Secondary Metabolites Production” in *Plants* [2].

The aim of this issue was to broaden the thematically presented works of the first volume, providing readers with up-to-date research on the production of plant secondary metabolites of different chemical types through the development of plant cells, tissues, and organs in diverse in vitro culture systems.

We have received six scientific research papers on approaches for secondary metabolite production based on the flexible tools of plant cell tissue and organ culture techniques.

In their work, Qahtan et al. [3] obtained efficient methods for the callus induction and plantlet regeneration of *Ruta chalepensis* L., a species traditionally utilized for the treatment of rheumatism, neuralgia, epilepsy, headache, intestinal worms, convulsion and menstrual disorders. The obtained ex vitro plant material showed higher levels of alkaloids, phenolics, flavonoids, tannins, and antioxidant activity as compared with the donor plant and the callus.

*Stevia rebaudiana* Bertoni is a plant gaining increasing popularity for the use of its diterpenoid steviol glycosides, 300 times sweeter than common table sugar. Steviol glycosides could be used as a sweetener substitute when sugar intake is undesirable due to health reasons. Sichanova et al. [4] carried out a stimulation of in vitro plant biomass formation and steviol glycoside enhancement through the treatment of *S. rebaudiana* shoot cultures with aminoacid silver nanofibers. The application of this agent also enhanced in vitro root formation, which plays a significant role in the micropropagation and potential adaptation of this species.

In their work, Marchant et al. [5] performed experiments on *Curcuma longa* L., testing the role of LED light together with a cultivation in a temporary immersion system bioreactor (TIS) and acclimatization in greenhouse conditions. The authors investigated the effect of the treatment on morphological characteristics; on polyphenol, tannin, flavonoid, reducing sugar and curcumin content; as well as on radical scavenging activity via DPPH, ORAC, and FRAP assays. The results showed that, during in vitro cultivation under TIS, the red/blue (RB) LED light spectrum promoted *C. longa* shoot proliferation, with the resulting seedlings exhibiting greater fresh weight and no signs of etiolation. In the acclimation phase, the RB spectrum increased phytochemicals, such as polyphenols, flavonoids, and reducing sugars, and boosted curcumin synthesis, but the antioxidant activity was not affected by the RB spectrum.

In their study, Danova et al. [6] compared the growth characteristics, hypericin productivity and processes occurring in the endogenous phytohormone pools of four *Hypericum* species belonging to different evolutionary stages. The study focused on the hypericin non-producing *H. calycinum* L. of the primitive *Ascyreia* section, hypericin-producing *H. perforatum* L., *H. tetrapterum* Fries, section *Hypericum*, and the evolutionarily most advanced *H. richeri* Vill. of the section *Drosocarpium*. It was established that both the evolutionary placement of the species, as well as the hypericin production capacity, interact closely with the endogenous phytohormones as a principal physiological parameter of the plants. The authors point out the possible hypothesis that hypericin productivity may have arisen in the evolution of *Hypericum* as a means of an environmental adaptation factor.

The cell culture of *Taraxacum officinale* (Weber) has been investigated by Martínez et al. [7], due to the long-lived application of the species as a traditional medicinal plant with anti-inflammatory, anti-carcinogenic, anti-rheumatic and other therapeutic effects. The inoculum size, subculture period and sucrose concentration were shown to affect α-amyrin and lupeol production. In their study on shoot cultures of the Balkan endemic *Sideritis scardica* Griseb., Danova et al. [8], established the effects of the application of either plant growth regulators (PGR) or activated charcoal (AC) to shoot cultures of the plant and the flavone/phenylethanoid ratio of the obtained plant material. The obtained results highlighted that shoot culture-derived plant material produced two phenylethanoids and five flavone glycosides not detected in the wild collected plant material. In addition, the two types of in vitro culture treatments led to the stimulation of either flavone glycosides or phenylethanoids in the in vitro cultivated plants. Thus, AC stimulated, to a higher extent, flavone glycosides’ accumulation, leading to an elevated flavone/phenylethanoid ratio, as compared with PGR treatments.
